# Prediction of Electric Power Production and Consumption for the CETATEA Building Using Neural Networks

**DOI:** 10.3390/s22166259

**Published:** 2022-08-20

**Authors:** Flaviu Turcu, Andrei Lazar, Vasile Rednic, Gabriel Rosca, Ciprian Zamfirescu, Emanuel Puschita

**Affiliations:** 1 National Institute for Research and Development of Isotopic and Molecular Technologies, 67-103 Donat Street, 400293 Cluj-Napoca, Romania; 2 Faculty of Physics, Babes-Bolyai University, 1 Kogalniceanu Street, 400084 Cluj-Napoca, Romania; 3 Communications Department, Technical University of Cluj-Napoca, 26-28 George Baritiu Street, 400027 Cluj-Napoca, Romania; 4 Department of Telecommunications, Politehnica University of Bucharest, 1-3, Iuliu Maniu Ave., 061071 Bucharest, Romania

**Keywords:** power prediction, neural networks, photovoltaic panels, real dataset, unified power production and consumption

## Abstract

Economic and social development is hardly influenced by electric power production and consumption. In this context of the energy supply pressure, energy production and consumption must be monitored and controlled in an intelligent way. Due to the availability of large data measurements, prediction algorithms based on neural networks are widely used in accurate power prediction. Firstly, the particularity of our work is represented by the size of the dataset consisting of 4 years of continuous real-time data measurements collected from the CETATEA photovoltaic power plant, a research site for renewable energies located in Cluj-Napoca, Romania. Secondly, the high granularity of the dataset with more than 4.2 million unified production and consumption power values recorded every 30 s guarantees the overall prediction accuracy of the system. Performance metrics used to evaluate the prediction accuracy are the mean bias error, the mean square error, the convergence time of the prediction system, the test performance, and the train mean performance. Test results indicate that the predicted unified electric power production and consumption closely resembles the unified electric power measured values.

## 1. Introduction

On a global scale, energy represents the key ingredient for economy development. Continuous increase in the energy demand [1] and the necessity to reduce pollution [2,3,4] make the renewable energy transition essential [5]. According to World Energy Outlook, in 2019 renewables had a share of global electricity generation of 23.2% [6]. Among wind, hydro-power, biomass, and geothermal, solar power is one of the most important renewable energy sources [7]. Unfortunately, the solar power at the ground level has the disadvantage of not being permanent and it is highly dependent on atmospheric parameters [8].

In the context of the overall energy supply pressure growing tension, both electric power production and consumption have increased progressively year by year. The randomness of solar power generation has three fundamental problems. Firstly, if too much power is generated and the grid cannot absorb it efficiently or store it in large batteries units, it becomes excess. Secondly, if there is not enough power generated and the grid cannot provide the requested energy, extra supply must be provided from the electric grid. Thirdly, if a system’s work is entirely dependent on the solar power generation, the owner cannot know if the system is available for a future application, or if there is enough time for the current running application to end successfully. Consequently, by providing a good estimation of both electric power generation and consumption (considering realistic limits and expectations), local power supply provisioning from other sources can be planned more efficiently.

In this context, the scope of this work is to provide a predictive method for unified electric power production and consumption for CETATEA, a building within the National Institute for Research and Development of Isotopic and Molecular Technologies, Cluj-Napoca, Romania. CETATEA is a research site for renewable energies (i.e., photovoltaic-solar energy conversion, concentrated solar energy, lead-acid batteries, wind energy, hydroelectric energy, energy recovery from mechanical vibration and electromagnetic pollution (electrosmog), thin layer thermoelectric transducers, unconventional treatments in microwave field, and fuel oil recovery from waste oils). Meantime, CETATEA is integrated with the national electric grid, and it is fully equipped with a photovoltaic (PV) system (i.e., photovoltaic panels, inverter, switch box, batteries, and electric meters).

When a predictive system is tested and evaluated in a real environment, it is recommended to consider that the system is as good as the training input data and its processing. This implies that the prediction increases proportionally to the distance and difference in the location. Most of the energy prediction systems have a direct dependency to the location and climate they were designed for. To provide a general predictive system outside of the area where the data was collected, there are two possible options: (1) either to have the entire system retrained with a new data set, or (2) to have the system working in a similar environment. In the first scenario, similar results can be obtained if the input data has the same parameters and size as the original dataset used. The size of the dataset must be at least the same or greater. In the second scenario, the trained system will provide similar results only if the new selected location has the same properties (i.e., the variables change in the same way as the weather).

The originality of this work consists of processing a set of real data collected from the CETATEA photovoltaic power plant. The unified electric power production and consumption prediction method proposed in this paper considers 4 years of real data measurements collected from January 2016 to December 2019 (i.e., 3 years for training and 1 year for testing). Unified electric power measured values are collected every 30 s.

The performance metrics used to evaluate the prediction systems are (1) the mean bias error (MBE), (2) the mean square error (MSE), (3) the convergence time of the prediction system, (4) the test performance (TEP), and (5) the train performance (TRP).

The main contributions of this work are as follows:

- Firstly, the accuracy of related theoretical prediction solutions is assessed by indicating the need to consider the application-specific particularities (i.e., number of datapoints in the frame time, neural network configuration in terms of epochs and regression methods, and the appropriate balance between training, testing, and computation time).
- Secondly, the impact of different configuration parameters of the proposed prediction system is evaluated. For the existing real electric power measurements dataset, the evaluation is performed by analyzing the impact of (1) the network dimensions and dataset size, (2) the error prediction, and (3) added noise on prediction accuracy.
- Thirdly, the access to a unified real dataset that contains all the information about the electric power production and all the information about the electric power consumption with a high resolution level (i.e., reported measurements every 30 s) is reported as a great need for an accurate prediction. The values for produced and consumed electric power are unified into a singular dataset, creating a complex system with a complex problem.
- Finally, the proposed electric power prediction method proves to be accurate in terms of train and test performances, MBE, and MSE, being capable of providing a convergence time required by long-term prediction. Test results indicated that the prediction model closely resembles the unified electric power measured values.

The remainder of this paper is organized as follows. Section 2 presents the state-of-art regarding prediction methods for electric power production and consumption. Section 3 provides a detailed description of unified power production and consumption chain at the CETATEA building. Meantime, in order to confirm if there is any power production drop of the PV farm, a thermal inspection of the solar panels is performed using a drone. Section 4 and Section 5 outline the implementation details of the prediction system and evaluate the impact of different configuration parameters on the prediction. Training results and validation of the power prediction model are presented in Section 6. Finally, Section 7 concludes the paper.

## 2. Literature Review

### 2.1. Electric Power Production using Photovoltaics Systems

Photovoltaic (PV) systems can produce electric power in every place (at a certain levels) being the ones with the highest solar potential among renewable energy resources [9]. Previous study showed that covering 16% of the world’s surface with 10% photovoltaic panels will produce two times the amount of energy compared to fossil fuels [10]. This extends the PV panels use and many PV systems were installed, starting from house use (few kW) to big farms (hundreds of MW) [11,12,13,14]. The increasing penetration of energy produced by PV systems into the power distribution grid has a negative effect by inducing voltage fluctuations due to the randomness of PV power production [15,16]. However, an accurate prediction of PV power production is of great help in finding the adequate methods of reducing the voltage fluctuations in the power distribution grids.

Solar PV and storage systems are classified into three types: (1) grid tied or grid direct PV system, (2) off grid PV system, and (3) grid/hybrid or grid interaction system with energy storage [17]. There are many types of PV panels produced today: mono-crystalline silicon, polycrystalline silicon, amorphous silicon, etc. Their efficiency is tested at standard conditions, 25 °C, and radiation level of 1000 W/m^2^ [18,19,20]. However, there are several factors that influence the PV panels’ efficiency and the amount of energy produced when the panels are installed in real working conditions. Evaluating different PV panel types, installed in different locations at different atmospheric conditions, stationary or mounted on one- and two-axis solar tracking systems, have been the subject of many studies [21,22,23,24,25,26,27,28,29,30,31,32,33,34,35,36].

### 2.2. Electric Power Production and Consumption Prediction

Yadav and Chandel [37] reviewed a series of promising results over various implementations of artificial neural networks with various combinations of input parameters. Such parameters are: the theoretical sunshine duration (TDSH), the measured sunshine duration (MDSH), the month, the maximum daily temperature, the monthly mean of theoretical sunshine duration (MTSH), the monthly mean of measured sunshine duration (SH), the extraterrestrial radiation (ER), etc. Most examples used either TDSH or MDSH, or a combination, alongside other parameters. The networks are implemented with a small number of hidden layers (1 or 2) and between 20 and 80 neurons per hidden layer. The results show a percentage root mean square error (RMSE %) between 5% and 14% with more than half of those values being around the 6% mark. Other examples mention as an indicator the absolute fraction of variance, the *R^2^* score, or the coefficient of determination as a percentage, with values ranging from as low as 88.9%, for a network with 3–11 inputs and only 6 neurons in the hidden layer, to values above 99% over a few examples, with most being above 95%.

Marzouq et al. [38] present a similar case for solar radiation prediction, with a review over 32 papers from various journals, over several years, with locations all over the world. The presented results show a series of low values, or percentages, for different error types, such as MBE or RMSE, with high correlation or coefficient of determination. The special mention in this case represents the small network size used in each example, with an average of 5–6 input nodes, 16 hidden layer nodes, and 23 total nodes per neural network, along with most networks using only 1 hidden layer.

Kuzlu et al. [39] use several explainable artificial intelligence tools to check which input parameters influence solar power prediction for a neural network the most. Out of a total of 13 input parameters, the ones with the highest importance marked by the tools are: surface solar radiation down (SSRD), the hour of the day (HOUR) and top net solar radiation (TSR), with the TCIW, surface thermal radiation down (STRD), and relative humidity (HUM %) showing in the following positions across different tools.

Across the literature, it is recommended to keep a 70–30 ratio between the training data and testing data [39]. In this case, it would require that the training data spans over 3 years while the testing data spans over 1 year, or less. While the testing data could be smaller, the training data must have a fixed span with 1 year periodicity to ensure an equal training development and to avoid biasing over specific months.

In machine learning, a different part during the training process is represented by the learning rate. This is represented by a set of constants that decrease the learning time (e.g., the number of iterations required to minimize the function), and the error. These constants are calculated using a cross-validation set, which represents a part of the data set that is not used during the training or for evaluating the system. For this purpose, a different way to split the data is 60–20–20, where 60% of the data set is used for training, 20% is used for as cross-validation, and the final 20% is used for testing.

In our work, the collected dataset of 4 years of real measurements is split in a 75–25 ratio for training–testing phases, while keeping the cyclic component in the prediction process. Furthermore, the 75% dataset used in training is split into a 75–25 ratio between actual training and validation process (i.e., 56.3–18.7% of the total dataset).

### 2.3. NARX-Based Model for PV Power Prediction

In this work, an NARX-based model (Nonlinear AutoregRessive with eXternal input) implemented in MatLab [40] is used for the PV power prediction. An NARX-based model is known for its application for chaotic time series analysis generated by nonlinear dynamic systems (e.g., the renewable energy sources) [41,42,43]. NARX is a partial recurrent neural network (RNN) as its memory is embedded into the network (i.e., network inputs include autoregressive terms apart from the external variables). In this way, the long-term dynamics (i.e., the cyclic property of the year, the day of the week, and the time of the day) captures the short-term dynamics of the system.

A large number of representative papers in the literature [41,42,43,44,45,46,47,48,49,50,51,52,53,54] show that the NARX neural network notably outperformed the other persistence models, and it is better than linear regression models. Accordingly, comparative test results analysis with other predicting methods in terms of similar statistical error measurements (i.e., mean bias error, mean absolute error, mean square error, root mean square error, normalized root mean squared error, and/or mean absolute percentage error) confirms NARX-based model prediction performances when it is used for renewable energy forecasting, as follows: (1) wind speed prediction [48,50,55,56], (2) solar irradiance prediction [45,51], (3) energy storage systems in photovoltaic installations [52], (4) output power prediction of the PV panels [53], electric power prediction [1], and (5) electrical load forecasting for buildings [47,48,57,58]. All these indicate that the NARX-based model has been implemented since the last decade for energy prediction issues and particularly it was widely adopted for forecasting the PV power output in relevant literature [41,42,43,47,49,53,57,58].

As the NARX-based prediction models have been widely adopted for forecasting the PV power output, the network dimensions were extensively tested to find the optimal NARX neural network configuration. In order to further improve the prediction accuracy, the performances of the NARX-based models were compared with two other prediction methods: (1) the error prediction approach and (2) the added noise over the original dataset approach.

With respect to previous published work in the area of forecasting energy prediction using an NARX-based approach, the following objective improvements are worthy of mention for the optimal network configuration:

- A high-resolution of the dataset (i.e., 30 s of continuous real-time measurements of unified electric power collected over a span of 4 years) up to 30 times the size of other reported datasets (i.e., from 5 to 15 min observations of PV power parameters over the course of 2, 3, or 4 years [41,43,49,57];
- Up to 20 times improvement in terms of prediction accuracy (i.e., MBE = −0.000089 and MSE = 0.0554) compared to reported network configurations [41,42,43,47,49].

## 3. CETATEA Photovoltaic Power Plant

In the context of renewable energy transition, the National Institute for Research and Development of Isotopic and Molecular Technologies (INCDTIM) form Cluj-Napoca, Romania, implemented a grant financed by the Sectorial Operational Program “Increasing economic competitiveness”, Investments for your future and co-financed by the European Regional Development Fund. Consequently, the CETATEA building was built, and the photovoltaic system was installed in 2015.

**Figure 1:**
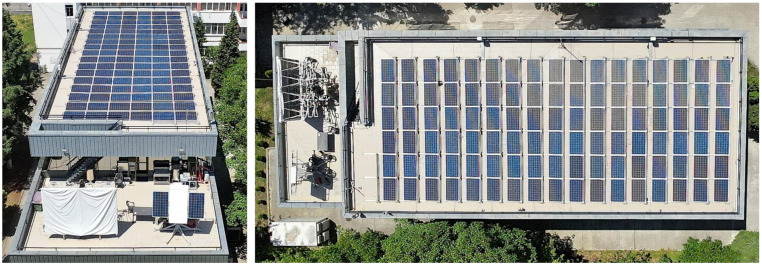
The CETATEA building at the National Institute for Research and Development of Isotopic and Molecular Technologies, Cluj-Napoca, Romania.

Figure 1 illustrates the CETATEA building and the photovoltaic farm.

### 3.1. CETATEA Photovoltaic System

The photovoltaic (PV) system is an island type, the maximum generated electric power is 25 kW. The produced energy is consumed locally but the excess is either transferred to a CellCube battery, that has a storage capacity of 40 kWh or is injected into the national power grid. The CETATEA photovoltaic system includes the following major blocks: (1) photovoltaic panels, (2) inverter, (3) switch box, (4) batteries, and (5) electric meters.

**Figure 2:**
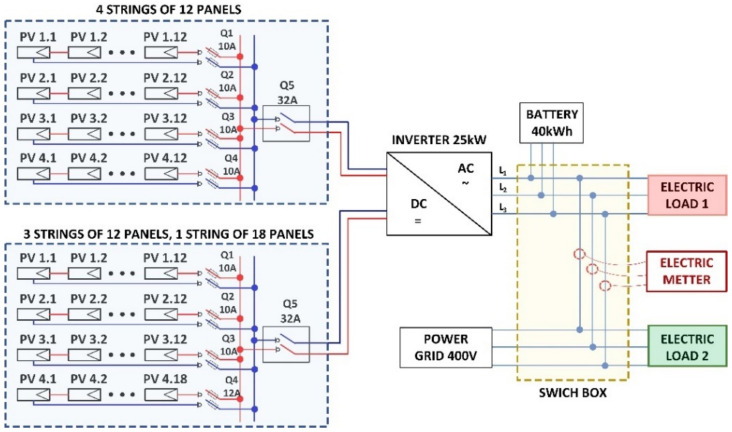
Electric schematic of the CETATEA photovoltaic system.

The electric connections diagram of the PV system is illustrated in Figure 2.

The PV farm consists of 102 PV panels. The PV panels are stationary having 22° southwest orientation and 10° from the horizontal. PV panels are grouped in 17 rows with 6 panels per row. Each two consecutive rows are connected in a series circuit except for the last three that are connected together (i.e., 7 string of 2 rows each, while the last string has 3 rows). Even the open circuit voltage of the 3 rows string is different compared to the 2 rows strings, the inverter has multistring capability with input voltage of up to 1000V. The solar panels involved in this study are polycrystalline type, JC245M-24/Bb model, produced by ReneSola Jiangsu Ltd. company [59].

**Figure 3:**
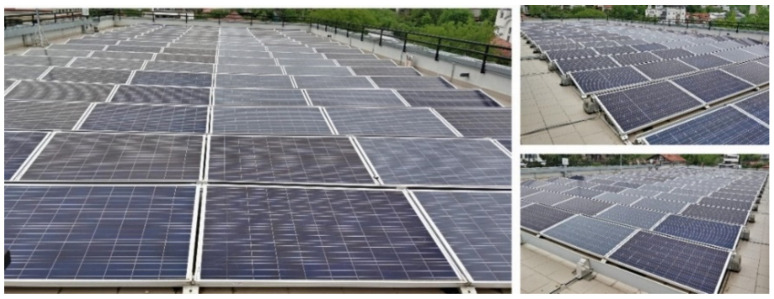
PV panels installed at the CETATEA building.

Figure 3 illustrates the PV panels installed at the CETATEA building.

Main characteristics of the PV panels are presented in Table 1.

The energy produced by the PV panels is transferred to an SMA Sunny Tripower 25,000 TL-30 inverter via two electric junction boxes. The inverter has multistring capability with DC input voltage of up to 1000 V and a maximum efficiency of 98.4%.

From the inverter the energy is transferred further to the consumer/storage unit/national power grid. The storage unit is a Vanadium redox flow battery FB 10–40. It has a power output of 10 kW and a storage capacity of 40 kWh.

**Figure 4:**
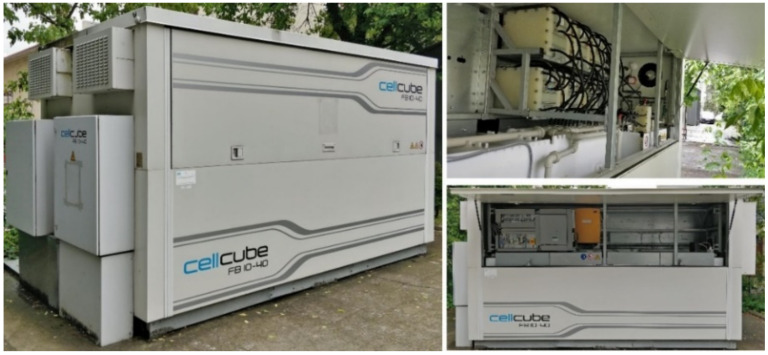
The storage unit attached to the PV system at the National Institute for Research and Development of Isotopic and Molecular Technologies, CETATEA building, Cluj-Napoca, Romania.

The storage unit is illustrated in Figure 4.

**Figure 5:**
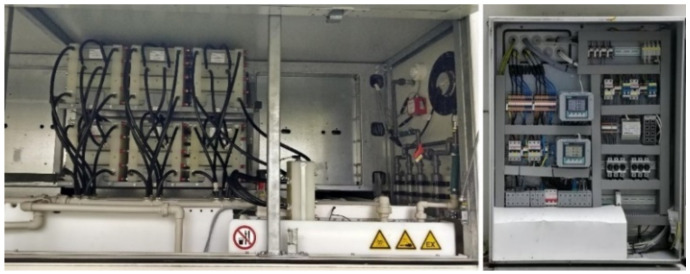
The batteries attached to the PV system including the digital meters of the monitoring panels.

The batteries and the associated control panels are presented in Figure 5.

There are nine digital electric meters that monitor the power produced by the PV system and the power consumed in different electric circuits of the CETATEA building. The digital electric meters provide information regarding (1) current intensity, (2) voltage, (3) active power, (4) reactive power, and (5) total energy yield. The dataset used in the present work includes the unified active power production and consumption values.

**Figure 6:**
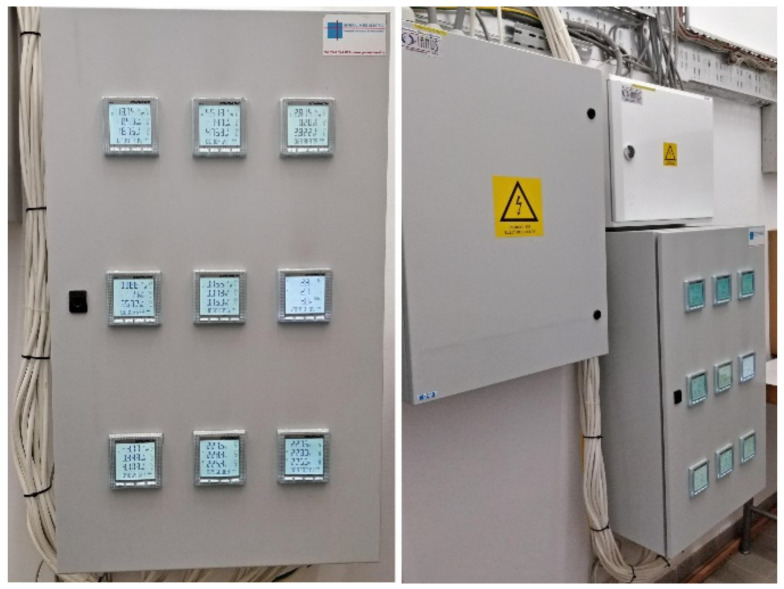
The monitoring panel of the PV system with nine digital electric meters.

The monitoring panel (i.e., digital electric meters) of the electric power produced/consumed locally/stored/injected into the national power grid is presented in Figure 6.

The electric meter computes the power transfer (P) between two parts of the electric circuit (as indicated in Figure 2). On the one hand there is the PV system, battery unit, and critical consumers (e.g., expressed as electric load 1), and on the other hand there is the power grid and non-critical consumers (e.g., expressed as electric load 2).

**Figure 7:**
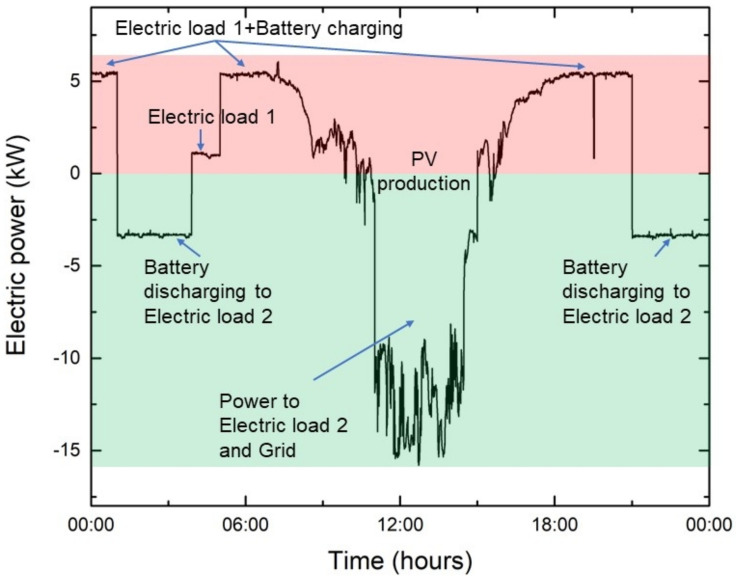
Unified electric power production and consumption over 24 h (1 March 2019).

The critical consumers require a permanent energy source and are directly connected to the battery unit (i.e., the battery unit operates in case of failures like an UPS unit). When the power is transferred from the power grid to the electric load 1 (critical consumers) or to charge the battery the *p* value is positive (please see red zone in Figure 7). Negative values of *p* indicate that the electric power is directly transferred from the PV system and/or battery unit to electric load 2 (non-critical consumers) and/or grid (please see green zone in Figure 7). Consequently, computed unified electric power produced and consumed is given by the following equation:

(1) $$P=P_{electric\;load\;1}-P_{PV}-P_{battery},$$

The *P_battery_* have positive values when the battery is in charging mode and negative values when the battery is in discharging mode.

The unified electric power production and consumption measurements over one day (1 March 2019) are presented in Figure 7.

### 3.2. Photovoltaic Panels Efficiency

Panel elements can break one by one over time, and the impact is felt over long intervals, directly into the power loss and financials. To confirm if there is any power production drop of the PV farm considered in our study, a thermal inspection of the panels was performed using a drone. This solution is reliable, precise using state-of-the-art equipment for the best quality data. We used an industrial-grade drone (i.e., model DJI M300 RTK, DJI, Shenzhen, China) equipped with a thermal vision specialized camera (i.e., Zenmuse H20N sensor with a 640 × 512 pixels image resolution for thermal images). It generates automatic reports with pinpointed anomalies and certain numbers for comparisons. The scene range is from −20 to 150 °C (High Gain) and from 0 to 500 °C (Low Gain). The spectral band is 8–14 μm and sensitivity (NETD) is ≤50 mK for aperture f/1.0.

Three types of solar cell anomalies were inspected: (1) solar cell anomalies, (2) diode defects, and (3) inverter anomalies.

Firstly, the PV panels were inspected for hot spots or solar cell anomalies. This type of anomaly is the most common. Shade on the module or a defective cell can change the module from power production to power consumption resulting in heating the cell which will show as a hot spot in a thermal image. Defective cells have a higher electrical resistance, which converts power into heat.

**Figure 8:**
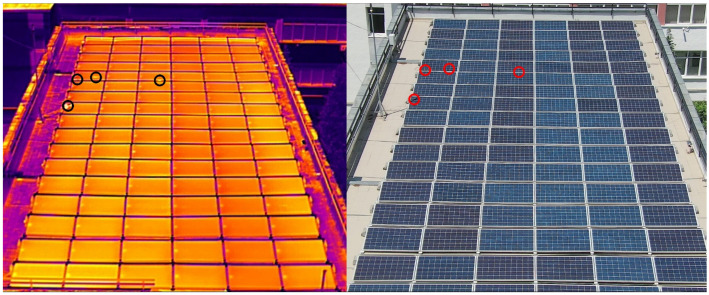
Thermal scanning using drone inspection displays the hot spot on the PV farm.

A comparison between RGB image and thermal view of the PV farm is presented in Figure 8.

The PV solar park configuration on the building rooftop is made of 102 panels with 60 cells each, obtaining a total of 6120 cells. We identified 3 panels with faulty cells caused by solar anomalies and a total of 8 cells were identified as faulty, which means 99.87% of the park is working properly. This kind of defect is hard to spot using traditional methods, that require an actual technician to manually check every panel and cell to pinpoint the faulty ones. By using the thermal vision camera, defective cells have a higher electrical resistance which converts power into heat. Hence, on the visual spectrum, the faulty cells will look brighter or darker. For very big areas of PV plants, artificial intelligence algorithms are used to count the faulty cells based on the input pictures captured with drones. In our case, a grid matrix is applied to inspect the 60 solar cells of each PV panel.

**Figure 9:**
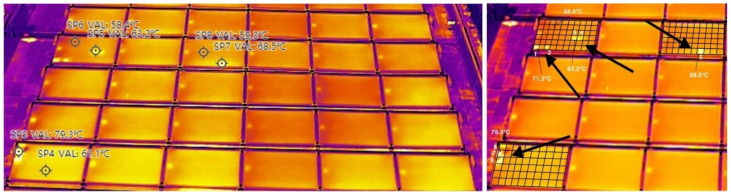
Hot spots indicating solar cell anomalies in the faulty panels (left side). Grid analysis on thermal images reveals the faulty cells as brighter spots in the visual spectrum (right side).

A closer view inspection reveals the 8 brighter faulty cells that are present on the PV farm (i.e., on the 3 solar panels), as illustrated in Figure 9.

There are reported temperature values of the solar cells between 55.6 (SP2) and 79.3 °C (SP3), as indicated in Table 2.

For a healthy PV panel, thermal scanning indicates the minimum working temperature of 58.6 °C and the maximum temperature of 60.9 °C. The highest temperature values in Table 2 (i.e., SP3 of 79.3 °C, SP5 of 63.2 °C, SP7 of 68.5 °C) confirm the hot spots on the 3 faulty panels (i.e., a total of 8 cells).

Secondly, the PV panels were inspected for diode defects and inverter anomalies. This are two other common types of failure of the solar cells. Statistically, taking into account the impact on energy production, the number of individual affected cells is higher than the diode defects. Additionally, one additional negative factor with high impact is caused by inverter anomalies. Those are rare and they affect the entire strings of modules. We did not identify those two types of defects or anomalies in our analysis.

Concluding, the thermal inspection indicates that the power production using the PV panels at CETAREA is efficient, and the produced power is similar to the estimated one.

## 4. Neural Network Approach for Energy Prediction

### 4.1. Neural Network Parameters

The dataset size and the network dimensions of any neural network impact its performance. Parameter optimization is meant to provide an accurate prediction of the unified energy production and energy consumption for the selected use case, namely the CETATEA building in Cluj-Napoca, Romania.

In our work, a set of tests were performed over a 4-year unified energy production and energy consumption dataset (i.e., real measurements collected from January 2016 to December 2019) to identify the optimal configuration of the neural network parameters. The monitoring system of the CETATEA building provides over 4.2 million unified produced and consumed power values in kilowatts (kW) (i.e., electric power measured values reported every 30 s). The electric power measured values reported for every 30 s results in a subset of 83.000–89.000 datapoints per month (e.g., the number of datapoints per month varies with the number of days in a month).

Considering the large size of the dataset, the actual training and testing is performed over a smaller number of unified energy measured values or points in the dataset. The dataset size may be adjusted by the value of the time division between the unified power measured values, but the user could also modify it by selecting a specific subset or by adding more data, if available. In this work, the time division indicates the time interval over which consecutive measurements are computed as a single average value. It controls the resolution of the dataset, namely the total number of points used in prediction.

The neural network has two dimensions which can be configured: (1) the length of the input delay and (2) the number of hidden layers.

The length of the input delay accounts for the number of previous points in the dataset used to predict the next one. The length of the input delay variation allows the user to monitor how a specific change in the value of the network dimensions impacts the overall prediction results. Hence, the larger the value of the input delay length gets, the better the result will be.

In our work, the unified power measurement values are averaged over a larger time division (i.e., the value of the time division is of minutes and hours) in order to reduce the dataset size, while still preserving year cyclicity information. Reducing the dataset size results in a faster training period (i.e., 10 or even 20 times faster). However, the scope of this initial set of tests is to evaluate the impact of dataset size and network dimensions on the overall power prediction, and to analyze the network parameter variation relative to each other (e.g., to check how a specific change in the network configuration parameters impacts the success of the prediction). The time division size determines the number of the data point measurements used in the training and validation sets. In our tests, the results will have the same dimension, representing an average over the selected time division.

To optimize the neural network performance, the selected values for the network dimension are meant to be on the smaller end (i.e., tens or hundreds of nodes, up to a few thousands), along with a reduced dataset to shorten the required training period (i.e., between tens of thousands to hundreds of thousands of values). This avoids both inconsistent results for a very small network sizes (i.e., networks with less than 10 nodes), and long computation training duration for larger networks (i.e., networks with tens of thousands of nodes). Nevertheless, the final set of tests (e.g., containing the optimal network configuration) was performed using the entire dataset.

### 4.2. Neural Network Configuration

According to the machine learning system description, the system is bound to have a good prediction only between the interval included within the training data. In this case, the system has a better energy prediction within a time frame, if that time frame had previous examples within our dataset. Hence, by a time frame we define a sliding window of a given length (i.e., a specified period of time of 3 years).

As a general overview, the prediction system is built using a neural network approach combining linear and logistic regression. The network contains several hidden layers that use a feedback loop to build on top of the next prediction. The logistic regression nodes are used to predict the chance of collecting solar power, while the linear regression nodes are used to predict the unified power production and consumption values.

**Figure 10:**
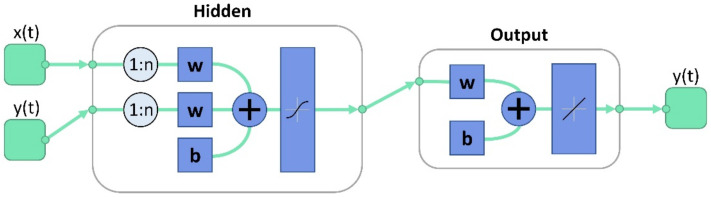
Conceptual design of the electric power prediction model developed in MatLab (x(t)—input data; y(t)—output data; w—weight; b—bias; 1:n—input delay length).

There are a several options that allow time series processing and prediction. The configuration used in our work is an NARX neural network, a nonlinear autoregressive neural network with exogenous input [8,60], as indicated in Figure 10.

The configuration parameters of the NARX-based neural network are: (1) the input delay length, (2) the number of hidden layers, and (3) the time division. They are configured before training phase. Network coefficients (also known as weights or the matrix parameters) are computed using the Scaled Conjugate Gradient algorithm. The training phase automatically closes when generalization stops improving, as indicated by an increase in the mean square error of the validation samples.

The network can be used in three specific ways: (1) open-loop, (2) closed-loop, and (3) multistep-ahead. In this study, the network is used in open-loop mode. As an open-loop system, the network can be referred to as a neural network with memory, where the memory represents an input delay buffer.

The input block represents a matrix formed by the input data (i.e., date and time), and the desired output, which must cover the same time frame, representing the input delay buffer. The size of the buffer represents the number of input data points used to predict a single value of the following point. This time frame is shifted by every iteration towards the next value until the end of the entire dataset.

The hidden layer consists of a set of weights through which the data is processed to improve the prediction. The hidden layer uses a sigmoid function. It is used in successive combinations and provides classification or regression [61]. The output block consists of a linear regression block that will predict the next value of the iteration. The trade-off is performed between the number of hidden nodes in each layer and the total number of hidden layers. Increasing the number of hidden nodes increases the system complexity, while improving the prediction. On the other hand, reducing the number of hidden nodes results in a more simplified system, while decreasing the training and computation time.

As a note, the data needs to be biased before it gets introduced in an NARX-based network because of how the sigmoid function behaves along the MatLab implementation of the linear regression output. On the one side, the unified produced and consumed measurements in the dataset are both positive and negative values (i.e., ranging between +10 and −15 kW; for details, see Figure 7). On the other side, the sigmoid function outputs values only between 0 and 1. By default, NARX model implementation is not able to correctly predict the negative values and considers them to be a prediction error. In this context, an initial bias is added to the entire dataset. It forces all input values to be strictly positive and match the output condition. In the end, the bias is subtracted from the output values, positioning the prediction back in the expected interval.

All NARX-based neural network simulations are performed in a MatLab environment using the Neural Network Time Series available in the Machine Learning and Deep Learning Toolbox (i.e., version R2020b, MathWorks, Natick, Massachusetts, United States of America). Detailed implementation of the NARX neural network structures is indicated in the neural network toolbox of MatLab [40].

**Figure 11:**
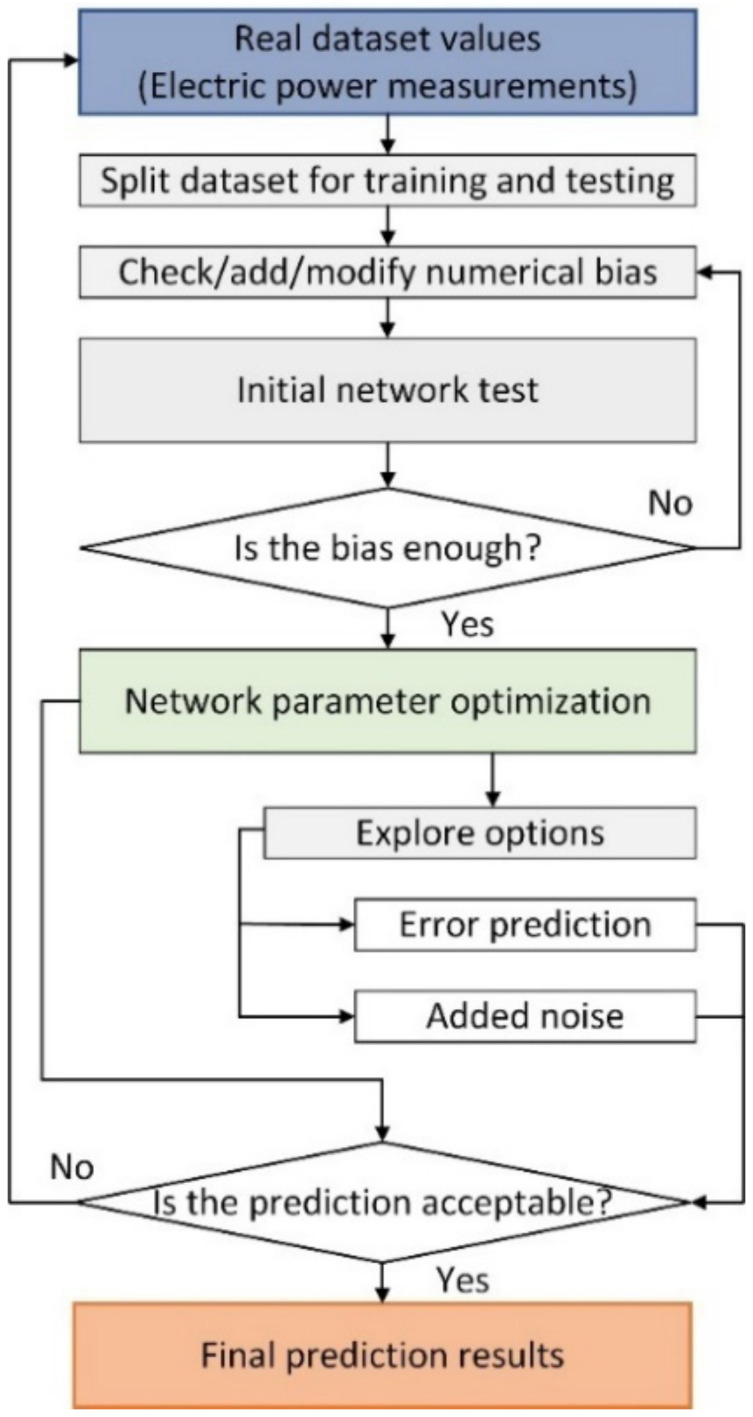
Unified electric power prediction workflow.

All the simulations were completed using a laptop with an intel i7 2.6 GHz processor and 16 GB of RAM. The energy prediction flowchart is illustrated in Figure 11.

### 4.3. Power Prediction Metrics

The prediction indices used in this work are as follows: (1) mean bias error (MBE), (2) mean square error (MSE), (3) convergence time of the prediction system, (4) test performance (TEP), and (5) train performance (TRP).

MBE indicates the system accuracy pointing out if any value out of bias exists in the system. MBE is used to evaluate long-term prediction and has a direct relation to the real value the system goes towards. It has a form of consistency over same-sized networks or same-sized datasets and is degraded when comparing different network or different dataset sizes. MSE is used to compare different network configurations running the same dataset. MSE is a good indicator for short-term prediction, correlating the width of the graph under which the errors are found. MSE degrades drastically if the initial dataset size is increased, showing a direct correlation in the overall network test performance.

Convergence time represents the minimum amount of time it takes for the network to train, while achieving an optimal result (e.g., the amount of time it takes for all the weights to be adjusted). This metric is a measure of system efficiency and indicates how fast the network computes the entire network weights for the overall power production and power consumption prediction measures. In some cases, the longer the network is trained (e.g., a higher convergence time), the lower the error becomes. However, after a certain amount of time, or a set of iterations, the network can overfit the training dataset. In this case the system tries to provide an answer far more complex than the real one. In this work, the convergence time is used to evaluate if the network training span is worth the error reduction.

The test/train performance functions return the error vs. number of iterations (i.e., epochs) lowest values during testing/training phases.

## 5. Neural Network Parameter Optimization

To improve the overall electric power prediction, the impact of the configuration parameters of the proposed neural network are evaluated. Consequently, for the existing energy measurements dataset, the effect of (1) network dimensions and dataset size, (2) error prediction, and (3) added noise is evaluated.

### 5.1. Impact of Dataset Size and Network Dimensions on the Overall Power Prediction

The first method to improve the overall power prediction of a neural network is to evaluate the impact of the network dimensions (i.e., input delay length and number of hidden layers) and the dataset size (i.e., time division). In the first step, the impact of the input delay length is evaluated.

Table 3 shows the network static configuration parameters.

The impact of increasing the input delay length is illustrated in Table 4.

The results indicate a noticeable increase in error for an input delay length of 60 points which decreases afterwards. The MBE and MSE values are significantly smaller for an input delay length of 10 points vs. an input delay length of 100 points, while maintaining similar values for performance. This is acknowledged for long-term prediction since the average over the entire prediction set might be very close.

Meanwhile, the results show a poor prediction for a small number of previous points since there is not enough information to correctly predict the next value. Therefore, a network with a small input delay length has a bad performance for short-term predictions. However, the length of the input delay does not impact the convergence time only for a large network size (i.e., input delay length above 100 points).

Secondly, the impact of the time division is evaluated. The time division impacts the number of data points used for training and testing phases.

Table 5 shows the network static configuration parameters.

The number of hidden layers is kept at 20 (consistent with the one mentioned in Table 3). The selected input delay length is of 40 points as it is the smaller value for which the network provides a similar MBE to the one at 140 points and MSE similar to the one at 100 points

The impact of time division variation is illustrated in Table 6.

In this experimental scenario, the decrease in the value of the time division (e.g., a larger number of data points), results in an improvement in the neural network in terms of train and test performances. While the average error seems to dip between the 60 and 30 min time divisions, the MSE decreases smoothly with the increased performance in the training and testing sides. The trade-off for test performance is the convergence time: the more data points a network is trained over, the longer the convergence time.

Thirdly, the impact of the number of hidden layers variation is evaluated for two network dimensions.

Table 7 shows the 1st network static parameters.

The impact of increasing the number of hidden layers for the 1st network configuration is illustrated in Table 8.

The results shows that the larger the number of hidden layers, the more errors are introduced into the prediction process. For a side comparison, a 2nd network is configured. The network dataset size is doubled (i.e., from 79,400 points to 158,800 points) by reducing the time division in half (i.e., from 20 min to 10 min), as indicated in Table 9.

The impact of increasing the number of hidden layers for the 2nd network configuration is presented in Table 10.

The results further demonstrate that there is a clear advantage in increasing the dataset size. It can be noticed that, for the same network size, the overall test performance is doubled (i.e., the value is decreased by half), while error decreases considerably. As the results indicate, for a number of 20 hidden layers, both MBE and MSE are lower independently of the network configuration. As a drawback, for 20 hidden layers, both MBE and MSE are several times lower.

Hence, by doubling the dataset size, the convergence time for the 2nd network configuration is about 3–5 times longer than the 1st one. This is due to a tweak in the network size, where the input delay length might be not sufficient to cover the information contained in a dataset twice as large.

### 5.2. Impact of Error Prediction on the Overall Energy Prediction

The second method investigated to improve the electric power prediction is the prediction of the actual error. In this case, the leftover error at the end of the initial prediction phase is used as input in the next iteration step.

**Figure 12:**
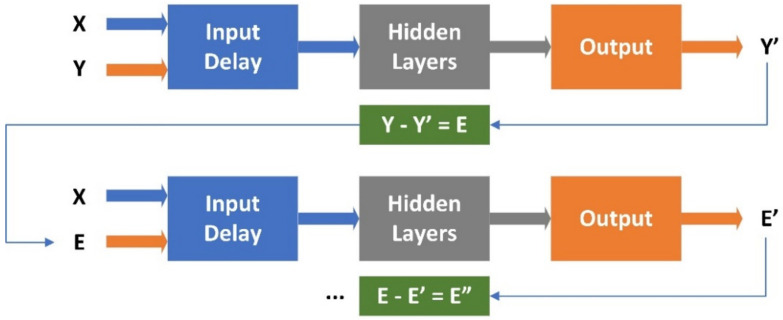
System configuration when evaluating the impact of error prediction (X—input data; Y—measured data; Y’—predicted output data; E—initial error; E’—leftover error predicted in the 1st iteration step; E”—leftover error predicted in the 2nd iteration step).

The network is retrained using the leftover error as its input, as indicated in Figure 12. After each step, the result is subtracted from the leftover. If successful, the leftover error should decrease after each step.

The impact of error prediction on the overall electric power prediction is evaluated in two experimental scenarios.

The neural network configuration parameters for two experimental test scenarios are indicated in Table 11.

Experimental prediction results when using the error prediction method in four iteration steps are presented in Table 12.

The results indicate that the prediction is not significantly improved by the error prediction method in terms of test performance, MBE, or MSE. In the case of the first experimental test scenario, the MBE barely decreases by 0.002 over four iteration steps, while the MSE decreases by 0.04. Meanwhile, the second experimental scenario does not indicate any improvement in the results in terms of test performance or error indexes over several steps. As expected, the total convergence time increases multiplicatively with the number of iteration steps because each iteration step for predicting the error took on average about the same time as the initial prediction step.

As a conclusion, the error prediction method does not improve the overall prediction due to a high number of measurement values in the dataset (i.e., unified electric power measurement values every 30 s). However, this method should still be considered in different scenarios or environments with a low-resolution dataset.

### 5.3. Impact of Added Noise on the Overall Electric Power Prediction

The third potential method to improve the prediction is to add noise over the original dataset. This method creates more data points in the training dataset, which is expected to improve the overall prediction.

In this scenario, the initial dataset is completed using data with added noise. In this case, the goal is not to reduce the error rate, but to improve the shape of the predicted signal to match the measurement variation in time. This approach is more suited for short-term prediction where time precision is more important than the actual value.

To evaluate the impact of added noise for the overall energy prediction, two types of random arrays are implemented. The first type of array contains random values in the interval [−0.05; +0.05], while the second type of array contains values in the interval [−0.1; +0.1]. The length of the random arrays matches the length of the original dataset, and the individual random arrays are different. Each newly added dataset is formed by the original dataset to which one of the two types of random arrays is added to (i.e., original data and noise).

**Figure 13:**
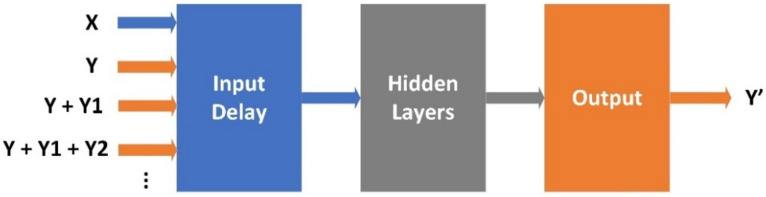
System configuration used to expand the size of the training dataset by adding random noise (X—input data; Y—measured data; Y’—predicted data; Y+Y1—added noise in the 1st iteration step; Y + Y1 + Y2—added noise in the 2nd iteration step).

The system configuration used to expand the size of the training dataset by adding noise is illustrated in Figure 13.

The network configuration parameters used in these tests are indicated in Table 13.

The impact of adding random noise over the initial training dataset is evaluated in two experimental scenarios: (1) the original dataset and three variations of added noise using the first random noise array, and (2) the original dataset, three variations of added noise using the first random noise array, and three variations of added noise using the second random noise array. Moreover, each of the experimental tests is performed using three different input delay length values.

The experimental test scenarios are performed in four iterative steps, as indicated in Table 14.

The results indicate that even the test performance has a slight decrease for specific network dimensions, the overall MSE prediction error increases with the added noise dataset. Moreover, by adding random noise to the initial dataset, in order to create more data points in the training dataset, the overall convergence time increases accordingly.

All in all, both error prediction and added noise methods do not improve the overall unified energy prediction for the current dataset of measurements.

Test results indicate that the initial dataset size has a very good resolution (i.e., unified electric power values measured every 30 s). Therefore, the most accurate prediction is provided by selecting the optimal network configuration in terms of time division, input delay length, and number of hidden layers.

## 6. Prediction of the Unified Electric Power Production and Consumption

Results show that the main factors that affect the predicted power output are the network dimensions and the length of the time division, given the existing dataset size. Error prediction and added noise do not significantly improve the prediction results, for a high-resolution dataset.

In this context, the feasibility of the proposed prediction method is validated using the complete dataset of unified electric power production and consumption collected over 4 years (i.e., collected from January 2016 to December 2019). The dataset is split in two subsets: (1) a time frame of 3 years used for training and validation (i.e., from January 2016 to December 2018), and (2) a time frame of 1 year for testing the performances of the prediction network (i.e., from January to December 2019). Each time frame duration is selected to provide a cyclic component in the prediction process. Consequently, the results are more consistent than running the simulations over an arbitrary time frame.

The proposed implementation of the NARX (Nonlinear AutoregRessive with eXternal input) neural network was evaluated in three different configurations. Configuration parameters for each network are illustrated in Table 15.

Predicted unified electric power production and consumption results for each network configuration are illustrated in Table 16.

**Figure 14:**
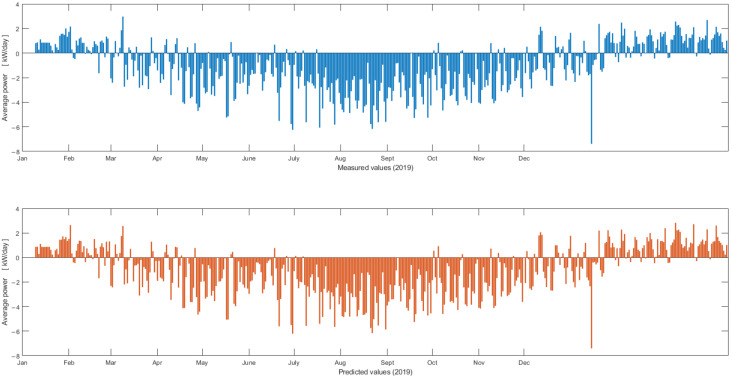
Measured (top—blue) and predicted (bottom—orange) unified electric power production and consumption over the year 2019 (one-day average electric power).

**Figure 15:**
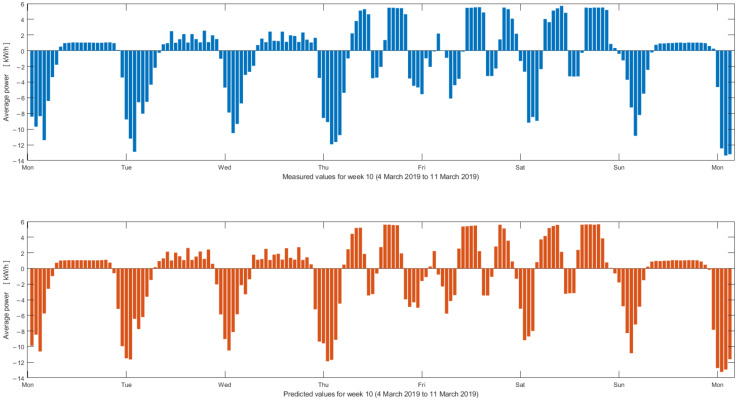
Measured (top—blue) and predicted (bottom—orange) unified electric power production and consumption over week 10 of 2019 (one-hour average electric power).

The analysis of the results in Table 16 indicate that the 2nd network configuration provides the best performance for the evaluated dataset. Testing results of the 2nd network configuration are presented in Figure 14 and Figure 15.

Figure 14 illustrates measured and predicted electric power values over the year 2019 (i.e., one-day average electric power).

Measured unified electric power production and consumption over the year 2019 (one-day average electric power) has mostly negative values (i.e., from February 2019 to November 2019). This corresponds to a higher PV energy production compared to the rest of the year. Test results confirm that the PV production is correlated with the yearly values of solar irradiance. Nevertheless, there are also exceptions (positive values) for this time interval that can be associated with the bad weather conditions.

Next, Figure 15 illustrates measured and predicted electric power values over week 10 of 2019 (i.e., 4 March 2019 to 11 March 2019).

Test results for one week at the hourly average power indicate that the predicted unified electric power production and consumption as well as the charging/discharging battery cycle (see details in Figure 7) closely follows the unified power measured values.

## 7. Conclusions

The scope of this work was to test and validate a predictive method for electric power production and consumption using an NARX neural network implementation. The feasibility of the proposed prediction method was validated using real electric power values collected over 4 years (i.e., from January 2016 to December 2019) from the CETATEA building. CETATEA is a building within the National Institute for Research and Development of Isotopic and Molecular Technologies, Cluj-Napoca, Romania, and was designed as a research site for renewable energies.

The major particularity that differentiates our work from other published papers is represented by (1) the size of the dataset (i.e., 4 years of continuous electric power measurements), and (2) the high-resolution of the dataset (i.e., unified electric power values measured every 30 s). With two measurements per minute, this research computes over 4.2 million distinct unified produced and consumed power measurement values. Consequently, a larger dataset size at a higher resolution for the measured values provides a clear improvement in terms of (3) the prediction accuracy. Moreover, the dataset integrates (4) continuous real power measurement values collected in the field, with no synthetic or artificially produced data.

The dataset is split in two subsets: (1) a time frame of 3 years used for training and validation (i.e., from January 2016 to December 2018) and (2) a time frame of one year used for testing the performances of the prediction network (i.e., from January to December 2019).

As the network was trained with real data collected by the CETATEA building, it is recommended that the proposed system configuration should be tested in similar conditions (e.g., same geographical latitude or on the opposite side of the equator but with a similar climate, similar position, and number of panels).

To select the appropriate network configuration parameters, various factors affecting the predicted electric power output were firstly analyzed. Hence, the impact of network dimensions and dataset size, error prediction, and added noise were evaluated.

The evaluation of different network dimension values indicates that the number of hidden layers and the input delay length require a high number of iterations to select the best set of parameters, which would yield a result closest to the real measurements.

Regarding the dataset size, the results confirm a clear improvement in prediction at a higher resolution for the measured values (i.e., the measured values in our dataset were collected every 30 s). However, if there are constraints for the training period (i.e., convergence time), a larger time division must be selected. Next, the evaluation of the convergence time confirms that the network dimensions and the size of the dataset directly impact the amount of time required to train the network and the total number of iterations.

To further optimize the neural network’s prediction, the impact of error prediction and added noise was evaluated in different experimental scenarios. In the case of error prediction, the system proved to be highly inefficient. This concludes that regardless of how accurate and precise a system is, randomness, or noise, is impossible to predict since it holds no information. In the case of added noise, the results indicate a slight improvement in terms of system performance without losing much on the error side. This concludes that the size of the added noise can be beneficial for the cases where the system’s complexity, respectively, the network size, is constrained by hardware. However, the impact of the added noise should be investigated as it substantially increases the required training period, and it also introduces the added noise as actual error.

All in all, the results indicated that both the error prediction and added noise methods do not improve the overall electric power prediction, due to a high resolution of the current dataset. Therefore, using the current dataset, a more accurate prediction is provided by selecting the optimal network configuration parameters in terms of the time division, input delay length, and number of hidden layers.

Finally, the feasibility of the proposed method was validated with the actual dataset of power generated and consumed. Testing results indicated that the predicted unified electric power production and consumption as well as the charging/discharging battery cycle) closely resembles the unified electric power measured values.

As future work, a solution to further improve the prediction performance is to extend the dataset size and the network dimensions, as well as the computational power.

## Figures and Tables

**Table 1:** PV panels’ main characteristics.

Parameter	Value
Maximum Power (Pmax)	245 W
Power Tolerance	0/+5W
Open Circuit Voltage (Voc)	37.3 V
Short Circuit Current (Isc)	8.73 A
Maximum Power Voltage (Vmp)	29.9 V
Maximum Power Current (Imp)	8.19 A
Maximum System Voltage	1000 VDC
Maximum Series Fuse Rating	20 A
Dimension (L × W × H)	1640 mm × 992 mm × 40 mm
Weight	19 kg

**Table 2:** Temperature measurements on different points of the PV systems.

Spot	Temperature Measured Value
SP1	62.0 °C
SP2	55.6 °C
SP3	79.3 °C
SP4	61.1 °C
SP5	63.2 °C
SP6	58.4 °C
SP7	68.5 °C
SP8	59.3 °C

**Table 3:** Network static configuration parameters (when evaluating the impact of input delay length).

Parameter	Value
Time division	20 min
Number of hidden layers	20
Number of training points	79,400
Number of test points	26,700

**Table 4:** The impact of input delay length variation.

Input Delay Length (Number of Previous Points)	10	40	60	100	140
Train performance	0.51	0.55	0.61	0.52	0.47
Test performance	0.82	0.89	0.98	0.87	0.82
MBE	0.0058	−0.0036	−0.086	0.16	−0.0039
MSE	0.42	0.72	0.82	0.7	0.68
Convergence time (seconds)	32	43	38	68	107

**Table 5:** Network static configuration parameters (when evaluating the impact of the time division).

Parameter	Value
Input delay length	40
Number of hidden layers	20

**Table 6:** The impact of the time division variation.

Time Division (Minutes)	60	45	30	20	15	5
Train points	26,400	35,000	52,900	79,400	105,800	317,600
Test points	8800	11,800	17,700	26,700	35,600	106,900
Train performance	0.82	0.73	0.61	0.48	0.47	0.32
Test performance	1.57	1.3	1	0.78	0.7	0.38
MBE	−0.21	0.0094	−0.22	−0.022	0.0083	0.0016
MSE	2.66	2.05	1.12	0.6	0.4	0.12
Convergence (seconds)	13	16	24	56	56	297

**Table 7:** The 1st network configuration values (when evaluating the impact of number of hidden layers).

Parameter	Value
Time division	20 min
Input delay length	40
Number of training points	79,400
Number of test points	26,700

**Table 8:** The impact of number of hidden layers variation (in the case of the 1st network configuration).

Number of Hidden Layers	5	10	20	40	60	80
Train performance	0.53	0.52	0.48	0.49	0.48	0.46
Test performance	0.82	0.8	0.78	0.81	0.76	0.76
MBE	−0.095	−0.053	−0.022	−0.083	−0.046	−0.098
MSE	0.34	0.62	0.6	0.66	0.64	0.71
Convergence (seconds)	45	38	56	77	135	289

**Table 9:** The 2nd network configuration values (when evaluating the impact of number of hidden layers).

Parameter	Value
Time division	10 min
Input delay length	40
Number of training points	158,800
Number of test points	53,400

**Table 10:** The impact of number of hidden layers variation (in the case of the 2nd network configuration).

Number of Hidden Layers	5	10	20	40	60	80
Train performance	0.343	0.43	0.38	0.322	0.314	0.312
Test performance	0.404	0.59	0.53	0.370	0.374	0.365
MBE	0.028	0.028	−0.003	−0.022	−0.028	−0.0056
MSE	0.149	0.158	0.119	0.106	0.133	0.128
Convergence (seconds)	153	153	322	488	902	1100

**Table 11:** Network parameters used to evaluate the impact of added noise.

Parameter	Experimental Scenario #1	Experimental Scenario #2
Time division	10 min	10 min
Input delay length	60	80
Number of hidden layers	40	60
Number of training points	158,800	158,800

**Table 12:** Unified power prediction results for the error prediction in four iteration steps.

Error Prediction Iteration Step		1	2	3	4
Experimental scenario #1	Test performance	0.410	0.393	0.392	0.395
	MBE	−0.0123	−0.0131	−0.0118	−0.010
	MSE	1.023	0.992	0.985	0.98
Experimental scenario #2	Test performance	0.371	0.373	0.375	0.381
	MBE	−0.0176	−0.0175	−0.0168	−0.0176
	MSE	0.947	0.943	0.941	0.948

**Table 13:** Network configuration parameters used to evaluate the impact of added noise.

Parameter	Value
Time division	20 min
Number of hidden layers	40
Number of training points (no randomness)	79,400
Number of test points	26,700

**Table 14:** Prediction results for added noise and input delay length variation.

Randomness		No Randomness	Experimental Scenario #1			Experimental Scenario #2		
Number of added sets		0	1	2	3	2	4	6
	Delay							
Test performance	40	0.82	0.75	0.75	0.73	0.76	0.86	0.78
	60	0.77	0.75	1.17	0.75	0.76	0.72	0.72
	80	0.76	0.77	1.44	0.75	0.75	0.84	0.72
MSE	40	0.5	0.54	0.79	0.58	0.72	0.65	0.62
	60	0.53	0.55	1.08	0.72	0.57	0.9	0.85
	80	0.55	0.66	0.88	0.67	0.61	0.89	0.81
Convergence (seconds)	40	59	212	502	647	495	1375	984
	60	86	253	154	719	325	1839	1869
	80	133	205	526	701	470	2077	2522

**Table 15:** Network configuration parameters.

Parameter	Network 1	Network 2	Network 3
Time division	1 min	1 min	1 min
Input delay length	60	80	100
Number of hidden layers	40	60	80
Number of training points	1,588,000	1,588,000	1,588,000
Number of test points	535,000	535,000	535,000

**Table 16:** Unified electric power production and consumption prediction results.

Network Configuration	Network 1	Network 2	Network 3
Train performance	0.1965	0.1886	0.1997
Test performance	0.1934	0.1858	0.1977
MBE	0.00105	−0.000089	−0.0019
MSE	0.0592	0.0554	0.0596
Convergence time (minutes)	51	135	180

## Data Availability

Not applicable.
